# Supplementary material for: In silico design of a multi-epitope vaccine against HPV16/18
Source: BMC Bioinformatics. 2022 Aug 2;23:311. doi: 10.1186/s12859-022-04784-x (PMC9344258; doi:10.1186/s12859-022-04784-x)
Supplement: Supplementary file 1 — Additional file1 Table S1: Results of CTL epitope prediction of E5 and E7 proteins from HPV16/18. Table S2: Results of HTL epitope prediction of E5 and E7 proteins from HPV16/18. Figure S1: Molecular docking of the selected CTL epitopes with HLA-A*02:01 molecule. Figure (A–F) represents the CTL epitopes E1-E6 respectively. The HLA-A*02:01 molecule is depicted in tan, and the CTL epitopes are depicted in blue. Figure S2: Molecular docking of the selected HTL epitopes with DRB1*01:01 molecule. Figure (A–I) represents the HTL epitopes E7-E15 respectively. The DRB1*01:01 molecule is shown in gray, and the HTL epitopes are shown in red. [file 12859_2022_4784_MOESM1_ESM.docx]

***In silico* design of a multi-epitope vaccine against HPV16/18**

Samira Sanami^a^, Mahmoud Rafieian-Kopaei^b^, Korosh Ashrafi Dehkordi^c^, Hamidreza Pazoki-Toroudi^d,e^, Fatemeh Azadegan-Dehkordi^f^, Gholam-Reza Mobini^f^, Morteza Alizadeh^g^, Muhammad Sadeqi Nezhad^h^, Maryam Ghasemi-Dehnoo^b^, Nader Bagheri^f^*

^a^ Department of Medical Biotechnology, School of Advanced Technologies, Shahrekord University of Medical Sciences, Shahrekord, Iran

^b^ Medical Plants Research Center, Basic Health Sciences Institute, Shahrekord University of Medical Sciences, Shahrekord, Iran

^c^ Department of Molecular Medicine, School of Advanced Technologies, Shahrekord University of Medical Sciences, Shahrekord, Iran

^d^ Physiology Research Center, Faculty of Medicine, Iran University of Medical Sciences, Tehran, Iran

^e^ Department of Physiology, Faculty of Medicine, Iran University of Medical Sciences, Tehran, Iran

^f^ Cellular and Molecular Research Center, Basic Health Sciences Institute, Shahrekord University of Medical Sciences, Shahrekord, Iran

^g^ Department of Tissue Engineering, School of Medicine, Shahroud University of Medical Sciences, Shahroud, Iran

^h^ Department of Clinical Laboratory Science, Young Researchers and Elites Club, Gorgan Branch, Islamic Azad University, Gorgan, Iran

***Corresponding author**: Cellular and Molecular Research Center, Basic Health Sciences Institute, Shahrekord University of Medical Sciences, Shahrekord, Iran. Tel: +98 9181731073; Fax: +98-3813330709. E-mail addresses: [n.bagheri1985@gmail.com](mailto:n.bagheri1985@gmail.com)

**Supplementary Table 1**

Results of CTL epitope prediction of E5 and E7 proteins from HPV16/18.

| **Epitope** | **Allels** | **E5 (HPV16)** | **E7 (HPV16)** | **E5 (HPV18)** | **E7 (HPV18)** | **VaxiJen score** | **Allergenicity** | **Toxicity** |
| --- | --- | --- | --- | --- | --- | --- | --- | --- |
| **FIVYIIFVY** | **A1, A3, A26, B58, B63** | ***** | **-** | **-** | **-** | **0.4718** | **No** | **No** |
| **FLIHTHARF** | **B58, A26, B8, B62** | ***** | **-** | **-** | **-** | **0.6381** | **No** | **No** |
| **YIIFVYIPL** | **A2, A26, B8, B39** | ***** | **-** | **-** | **-** | **0.5795** | **No** | **No** |
| **YTSLIILVL** | **A1, A2, B8, B39, B58, B69** | ***** | **-** | **-** | **-** | **0.6175** | **No** | **No** |
| **RAHYNIVTF** | **A24, B7, B8, B58, B62** | **-** | ***** | **-** | **-** | **0.5919** | **No** | **No** |
| ATAFTVYVF | B58, A1, A24, B62 | - | - | * | - | -0.0642 | No | No |
| **YAWVLVFVY** | **B8, B58, A1, B62** | **-** | **-** | ***** | **-** | **0.4484** | **No** | **No** |
| FQQLFLNTL | A2, B44, B62, B8, B39 | - | - | - | * | -0.6011 | No | No |
| RAEPQRHTL | B8, B7, B39, A26, B62 | - | - | - | * | -0.2584 | Yes | No |

The selected epitopes have been shown in bold.

**Supplementary Table 2**

Results of HTL epitope prediction of E5 and E7 proteins from HPV16/18.

| **Epitope** | **Allels** | **E5 (HPV16)** | **E7 (HPV16)** | **E5 (HPV18)** | **E7 (HPV18)** | **VaxiJen score** | **Allergenicity** | **Toxicity** | **IFN-γ -inducing** |
| --- | --- | --- | --- | --- | --- | --- | --- | --- | --- |
| **FVYIPLFLIHTHARF** | **HLA-DRB10401, HLA-DRB10701, HLA-DPA10201-DPB10101, HLA-DPA10103-HLA-DPB10301_DPB10401, HLA-DRB11501, HLA-DRB50101, HLA-DPA10301-DPB10402, HLA-DRB10101** | ***** | **-** | **-** | **-** | **0.548** | **No** | **No** | **Positive** |
| IPLFLIHTHARFLIT | HLA-DRB10401, HLA-DRB11101, HLA-DRB11302, HLA-DRB10701, HLA-DRB50101, HLA-DRB11501, HLA-DPA10103-DPB10201, HLA-DRB10101 | * | - | - | - | 0.3569 | No | No | Positive |
| **IRPLLLSVSTYTSLI** | **HLA-DRB10405, HLA-DRB11501, HLA-DRB10701, HLA-DRB10401, HLA-DPA10103-DPB10201, HLA-DRB10101, HLA-DRB10404** | ***** | **-** | **-** | **-** | **0.54** | **No** | **No** | **Positive** |
| LLLWITAASAFRCFI | HLA-DRB10901, HLA-DRB50101, HLA-DQA10501-DQB10301, HLA-DRB10101, HLA-DQA10102-DQB10602, HLA-DRB10404, HLA-DRB10701 | * | - | - | - | -0.0133 | No | No | Positive |
| LVLLLWITAASAFRC | HLA-DRB10404, HLA-DRB10101, HLA-DRB10901, HLA-DQA10501-DQB10301, HLA-DRB10701, HLA-DRB50101, HLA-DQA10102-DQB10602 | * | - | - | - | 0.3969 | No | No | Negative |
| **RPLLLSVSTYTSLII** | **HLA-DRB10405, HLA-DRB11501, HLA-DPA10103-DPB10201, HLA-DRB10401, HLA-DRB10701, HLA-DRB10404, HLA-DRB10101** | ***** | **-** | **-** | **-** | **0.4983** | **No** | **No** | **Positive** |
| VLLLWITAASAFRCF | HLA-DQA10501-DQB10301, HLA-DRB10901, HLA-DRB10101, HLA-DRB50101, HLA-DRB10404, HLA-DQA10102-DQB10602, HLA-DRB10701 | * | - | - | - | 0.2937 | No | No | Negative |
| YIPLFLIHTHARFLI | HLA-DRB10701, HLA-DRB10401, HLA-DRB11101, HLA-DRB11302, HLA-DRB50101, HLA-DRB11501, HLA-DRB10101 | * | - | - | - | 0.3244 | No | No | Positive |
| **DSTLRLCVQSTHVDI** | **HLA-DRB10404, HLA-DRB10701, HLA-DRB40101** | **-** | ***** | **-** | **-** | **0.5514** | **No** | **No** | **Positive** |
| STLRLCVQSTHVDIR | HLA-DRB10101, HLA-DRB10404, HLA-DRB10701, HLA-DRB40101 | - | * | - | - | 0.8539 | Yes | No | Positive |
| CDSTLRLCVQSTHVD | HLA-DRB10404, HLA-DRB10701, HLA-DRB40101 | - | * | - | - | 0.4818 | Yes | No | Positive |
| LRLCVQSTHVDIRTL | HLA-DRB10404, HLA-DRB10701, HLA-DRB40101 | - | * | - | - | 0.7711 | Yes | No | Positive |
| TLRLCVQSTHVDIRT | HLA-DRB10404, HLA-DRB10701, HLA-DRB40101 | - | * | - | - | 0.7219 | Yes | No | Positive |
| FVYIVVITSPATAFT | HLA-DRB10101, HLA-DRB10404, HLA-DRB10405, HLA-DRB10701, HLA-DRB10901, HLA-DRB11101, HLA-DRB11302, HLA-DRB11501 | - | - | * | - | 0.3011 | No | No | Negative |
| VFVYIVVITSPATAF | HLA-DRB10101, HLA-DRB10404, HLA-DRB10405, HLA-DRB10701, HLA-DRB10901, HLA-DRB11101, HLA-DRB11302, HLA-DRB11501 | - | - | * | - | 0.0412 | No | No | Positive |
| VYIVVITSPATAFTV | HLA-DRB10101, HLA-DRB10404, HLA-DRB10405, HLA-DRB10701, HLA-DRB10901, HLA-DRB11101, HLA-DRB11302, HLA-DRB11501, HLA-DQA10501-DQB10301 | - | - | * | - | 0.239 | No | No | Negative |
| YIVVITSPATAFTVY | HLA-DRB10101, HLA-DRB104, HLA-DRB10701, HLA-DRB10901, HLA-DRB11101, HLA-DRB11302, HLA-DRB11501, HLA-DQA10501-DQB1030 | - | - | * | - | 0.1691 | No | No | Negative |
| **AFTVYVFCFLLPMLL** | **HLA-DRB10101, HLA-DPA10103-DPB10401, HLA-DPA10103-DPB10201, HLA-DPA10201-DPB10101, HLA-DPA10103-HLA-DPB10301_DPB10401, HLA-DPA10301-DPB10402** | **-** | **-** | ***** | **-** | **0.4578** | **No** | **No** | **Positive** |
| ADDLRAFQQLFLNTL | HLA-DRB10101, HLA-DRB10405, HLA-DRB10701, HLA-DRB40101, HLA-DRB50101, HLA-DPA10103-DPB10401, HLA-DPA10103-DPB10201, HLA-DPA10201-DPB10101, HLA-DPA10103-HLA-DPB10301_DPB10401, HLA-DPA10103-HLA-DPB10301_DPB10401 | - | - | - | * | -0.4447 | Yes | No | Negative |
| **DGVNHQHLPARRAEP** | **HLA-DRB10101, HLA-DRB10701, HLA-DPA10103-DPB10401, HLA-DPA10103-HLA-DPB10301_DPB10401** | **-** | **-** | **-** | ***** | **0.7849** | **No** | **No** | **Positive** |
| AFQQLFLNTLSFVCP | HLA-DRB10101, HLA-DRB10401, HLA-DRB10405, HLA-DPA10103-DPB10401, HLA-DPA10103-DPB10201, HLA-DPA10201-DPB10101, HLA-DPA10103-HLA-DPB10301_DPB10401, HLA-DPA10103-HLA-DPB10301_DPB10401, HLA-DPA10301-DPB10402 | - | - | - | * | 0.339 | Yes | No | Negative |
| DDLRAFQQLFLNTLS | HLA-DRB10101, HLA-DRB10401, HLA-DRB10405, HLA-DRB10701, HLA-DRB40101, HLA-DRB50101, HLA-DPA10103-DPB10401, HLA-DPA10103-DPB10201, HLA-DPA10201-DPB10101, HLA-DPA10103-HLA-DPB10301_DPB10401, HLA-DPA10103-HLA-DPB10301_DPB10401, HLA-DPA10301-DPB10402 | - | - | - | * | -0.4063 | No | No | Negative |
| **EIDGVNHQHLPARRA** | **HLA-DRB10101, HLA-DRB10405, HLA-DPA10301-DPB10402** | **-** | **-** | **-** | ***** | **0.6817** | **No** | **No** | **Positive** |
| DLRAFQQLFLNTLSF | HLA-DRB10101, HLA-DRB10401, HLA-DRB10405, HLA-DRB10701, HLA-DRB40101, HLA-DRB50101, HLA-DPA10103-DPB10401, HLA-DPA10103-DPB10201, HLA-DPA10201-DPB10101, HLA-DPA10103-HLA-DPB10301_DPB10401, HLA-DPA10103-HLA-DPB10301_DPB10401 | - | - | - | * | 0.0101 | No | No | Negative |
| FQQLFLNTLSFVCPW | HLA-DRB10101, HLA-DPA10103-DPB10401, HLA-DPA10103-DPB10201, HLA-DPA10201-DPB10101, HLA-DPA10103-HLA-DPB10301_DPB10401, HLA-DPA10103-HLA-DPB10301_DPB10401, HLA-DPA10301-DPB10402 | - | - | - | * | 0.2437 | No | No | Negative |
| LRAFQQLFLNTLSFV | HLA-DRB10101, HLA-DRB10401, HLA-DRB10405, HLA-DRB10701, HLA-DRB40101, HLA-DRB50101, HLA-DPA10103-DPB10401, HLA-DPA10103-DPB10201, HLA-DPA10201-DPB10101, HLA-DPA10103-HLA-DPB10301_DPB10401, HLA-DPA10103-HLA-DPB10301_DPB10401, HLA-DPA10301-DPB10402 | - | - | - | * | -0.1428 | No | No | Negative |
| RAFQQLFLNTLSFVC | HLA-DRB10101, HLA-DRB10401, HLA-DRB10405, HLA-DRB50101, HLA-DPA10103-DPB10401, HLA-DPA10103-DPB10201, HLA-DPA10201-DPB10101, HLA-DPA10103-HLA-DPB10301_DPB10401, HLA-DPA10103-HLA-DPB10301_DPB10401, HLA-DPA10301-DPB10402 | - | - | - | * | 0.164 | Yes | No | Negative |
| **GVNHQHLPARRAEPQ** | **HLA-DRB10101, HLA-DPA10103-DPB10201, HLA-DRB10701** | **-** | **-** | **-** | ***** | **0.9309** | **No** | **No** | **Positive** |
| SADDLRAFQQLFLNT | HLA-DRB10701, HLA-DRB40101, HLA-DPA10103-DPB10401, HLA-DPA10103-DPB10201, HLA-DPA10201-DPB10101, HLA-DPA10301-DPB10402 | - | - | - | * | -0.2429 | No | No | Negative |
| **IDGVNHQHLPARRAE** | **HLA-DRB10101, HLA-DPA10103-DPB10401, HLA-DQA10102-DQB10602, HLA-DRB10101** | **-** | **-** | **-** | ***** | **0.7585** | **No** | **No** | **Positive** |

The selected epitopes have been shown in bold.


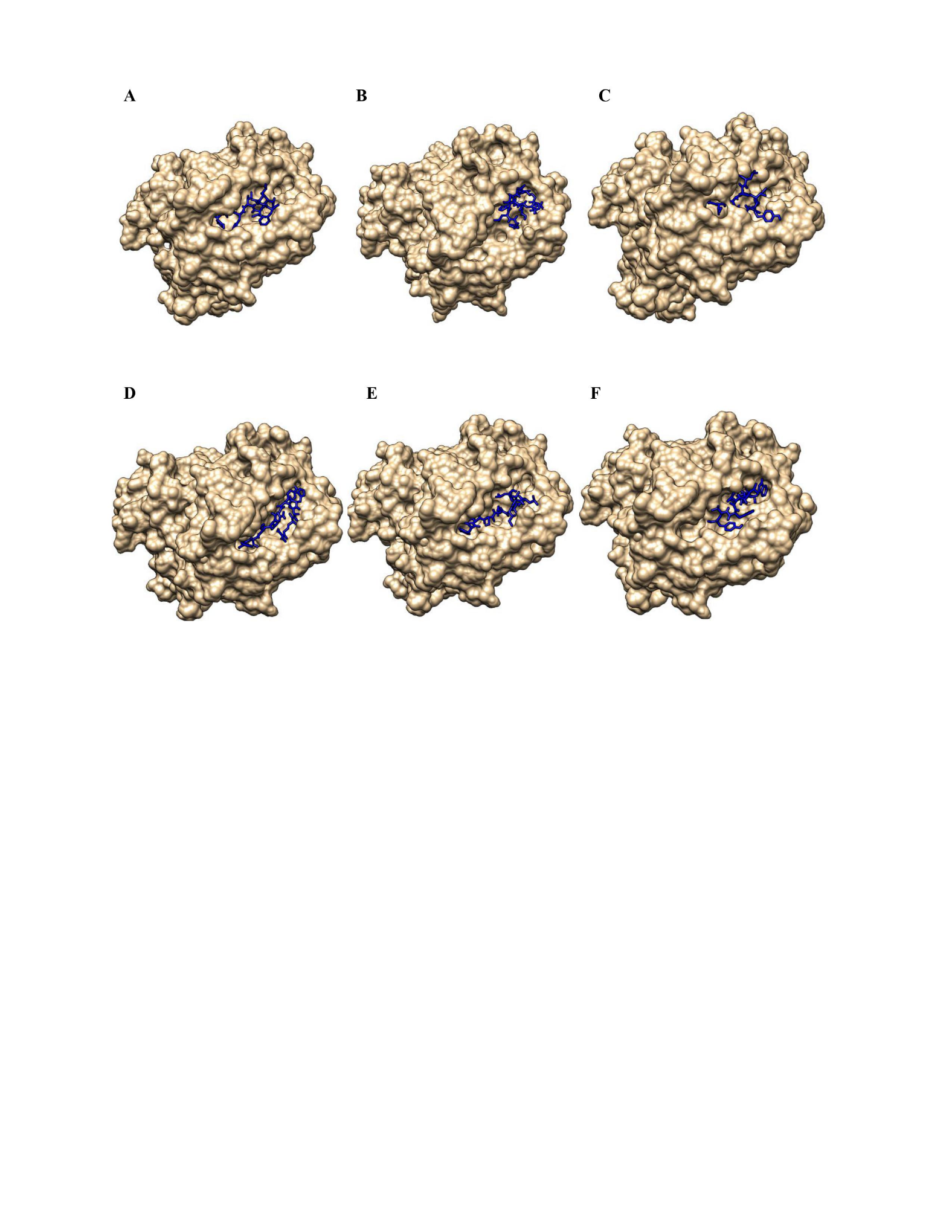


**Supplementary Fig. 1.** Molecular docking of the selected CTL epitopes with HLA-A*02:01 molecule. Figure (A–F) represents the CTL epitopes E1-E6 respectively. The HLA-A*02:01 molecule is depicted in tan, and the CTL epitopes are depicted in blue.


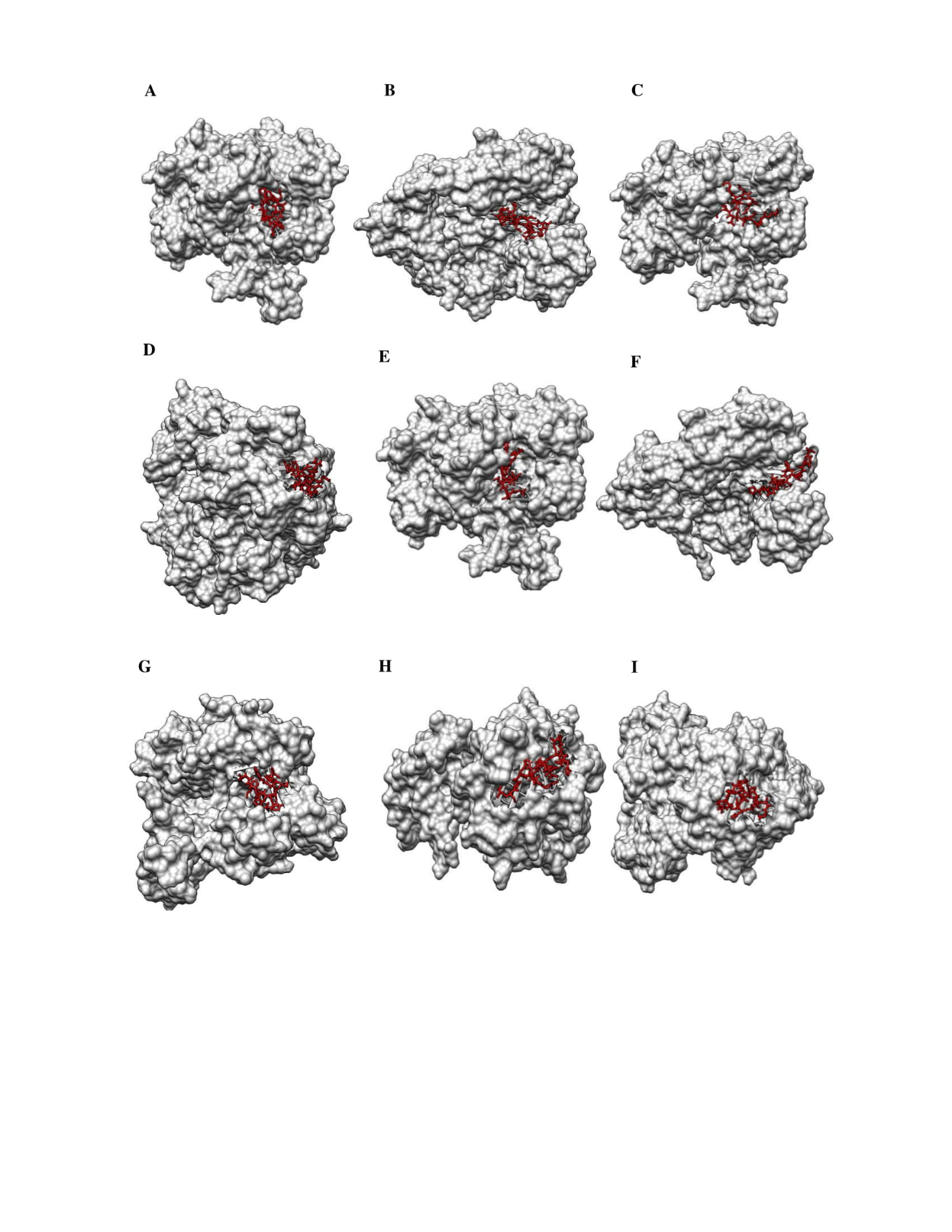


**Supplementary Fig. 2.** Molecular docking of the selected HTL epitopes with DRB1*01:01 molecule. Figure (A–I) represents the HTL epitopes E7-E15 respectively. The DRB1*01:01 molecule is shown in gray, and the HTL epitopes are shown in red.
